# The Effect of Systemic and Regional Use of Magnesium Sulfate on Postoperative Tramadol Consumption in Lumbar Disc Surgery

**DOI:** 10.1155/2016/3216246

**Published:** 2016-01-28

**Authors:** Melek Demiroglu, Canan Ün, Dilsen Hatice Ornek, Oya Kıcı, Ali Erdem Yıldırım, Eyup Horasanlı, Semih Başkan, Emel Fikir, Mehmet Gamli, Bayazit Dikmen

**Affiliations:** ^1^Ankara Numune Training and Research Hospital, Anesthesiology Department, Ankara, Turkey; ^2^Ankara Numune Training and Research Hospital, Neurosurgery Department, Ankara, Turkey; ^3^Ankara Yıldırım Beyazit University Medical School Anesthesiology Department, Ankara, Turkey; ^4^Ankara Gazi University Medical School Anesthesiology Department, Ankara, Turkey

## Abstract

*Aim.* To investigate the effect of magnesium administered to the operative region muscle and administered systemically on postoperative analgesia consumption after lumbar disc surgery.* Material and Method.* The study included a total of 75 ASA I-II patients aged 18–65 years. The patients were randomly allocated into 1 of 3 groups of 25: the Intravenous (IV) Group, the Intramuscular (IM) Group, and the Control (C) Group. At the stage of suturing the surgical incision site, the IV Group received 50 mg/kg MgSO_4_ intravenously in 150 mL saline within 30 mins. In the IM Group, 50 mg/kg MgSO_4_ in 30 mL saline was injected intramuscularly into the paraspinal muscles. In Group C, 30 mL saline was injected intramuscularly into the paraspinal muscles. After operation patients in all 3 groups were given 100 mg tramadol and 10 mg metoclopramide and tramadol solution was started intravenously through a patient-controlled analgesia device. Hemodynamic changes, demographic data, duration of anesthesia and surgery, pain scores (NRS), the Ramsay sedation score (RSS), the amount of analgesia consumed, nausea- vomiting, and potential side effects were recorded.* Results.* No difference was observed between the groups. Nausea and vomiting side effects occurred at a rate of 36% in Group C, which was a significantly higher rate compared to the other groups (*p* < 0.05). Tramadol consumption in the IM Group was found to be significantly lower than in the other groups (*p* < 0.05).* Conclusion.* Magnesium applied to the operative region was found to be more effective on postoperative analgesia than systemically administered magnesium.

## 1. Introduction

The management of pain following surgery and general anesthesia is extremely important. Opioids are widely used in postoperative analgesia but have side effects including respiratory depression, nausea, vomiting, hypotension, tachycardia, sweating, and itching [1, 2]. Therefore, medications and adjuvant agents to reduce the need for opioids have become widely used.

Magnesium, which is the fourth most abundant cation in the human body, has an antinociceptive effect and plays an important role in neural communication [3, 4]. While it blocks voltage-dependent ion channels, it is also a natural calcium antagonist and controls calcium's entrance into cells [5]. The *α*2*δ* subunit of voltage-dependent calcium channels is responsible, in part, for pain; magnesium has been shown to have an affinity for this receptor [6]. Magnesium is known to antagonize the expression of inflammatory mediators (histamine, serotonin, and cytokines) in peripheral tissues [7].

In addition, magnesium is a noncompetitive N-methyl-D-aspartate (NMDA) receptor antagonist. In the central nociceptive communication of acute pain, NMDA receptors play a major role in modulation and sensitization [8, 9]. In addition to the central localization of NMDA receptors, receptors in the periphery (muscle, skin, and knee joint) have been shown to play a role in the transport of pain stimuli [10, 11]. NMDA receptor antagonists give opioids the potential to have an analgesic effect.

It has been suggested that the decrease in plasma magnesium levels during major surgical interventions could not be sufficiently counteracted by using magnesium-containing solutions [12]. There are, however, studies showing that the systemic administration of magnesium (MgSO_4_) has reduced postoperative opioid consumption [13, 14]. It has also been reported that magnesium administered to the surgical area reduced postoperative analgesia consumption [15].

The aim of this study was to compare the effects of systemic administration of MgSO_4_ and intramuscular administration of MgSO_4_ to the paraspinal muscles on postoperative tramadol consumption after lumbar disc herniation surgery.

## 2. Method

Approval for the study was granted by the Local Ethics Committee and informed consent was obtained from all patients. The study included 75 ASA I-II patients aged 18–65 years. Patients were excluded if they had severe cardiovascular, hepatic, renal, or neuropsychiatric disease, if they had known sensitivity to the medications used in the study, or if they had a history of long-term opioid use. During the preoperative evaluation, the patients were informed about the Numeric Pain Rating Scale (NRS) and the patient-controlled analgesia (PCA) device. The patients were randomly allocated into 1 of 3 groups of 25 patients each: the Intravenous (IV) Group, the Intramuscular (IM) Group, and the Control (C) Group.

When the patients were admitted to the operating room, ECG, noninvasive arterial pressure, heart rate, and peripheral oxygen saturation monitors were applied. A 20 G cannula was placed into a peripheral vein on the back of the hand and crystalloid infusion was started at 5–10 mL/kg/hour. All necessary fluid and blood replacement was provided. In induction, both groups were given 6 mg/kg thiopental sodium, 2 mcg/kg fentanyl, 0.1 mg/kg vecuronium bromide, and 1 mg/kg lidocaine hydrochloride. Anesthesia was maintained with 50% N_2_O, 50% O_2_, and sevoflurane (MAC 1, 2) at 2 L/min.

At the time of suturing the surgical incision, 50 mg/kg MgSO_4_ in 150 mL saline was applied intravenously over 30 mins in the IV Group, while 50 mg/kg MgSO_4_ in 30 mL saline was injected intramuscularly to the paraspinal muscles in the IM Group and 30 mL saline was injected intramuscularly to the paraspinal muscles in the C Group. In the perioperative period, no other medication or additional dose that could affect the study method or the mechanism of the analgesic medication was used. In addition, when the incision site was closed, all three groups were given 100 mg tramadol and 10 mg metoclopramide intravenously. For decurarization, 0.01 mg/kg atropine and 0.05 mg/kg neostigmine were administered.

In the recovery room at the end of the operation, when the patients could correctly state their own date of birth, they were deemed conscious and had the PCA attached with tramadol solution prepared in the standard manner (8 mg tramadol/mL) (PCA bolus dosage: 16 mg, locked time: 10 mins, no infusion). In the postoperative period, patients with NRS > 6 were given intramuscular diclofenac sodium as additional analgesia. Patients with complaints of nausea and vomiting were given 10 mg metoclopramide.

All patients in all groups were evaluated in the recovery room at 5, 15, 30, 45, and 60 mins postoperatively and then on the ward at 4, 8, 12, and 24 hours for the following parameters: hemodynamic changes, demographic data, duration of surgery and anesthesia, pain scores (NRS 0–10: 0 = no pain and 10 = intolerable pain), RSS (Ramsay sedation score: 1 = agitated and anxious; 2 = calm, cooperative, and oriented; 3 = sleeping and cooperative with verbal instructions; 4 = sleeping, having glabellar reflex with mild tapping, or easily cooperating with verbal instructions; 5 = sleeping, having glabellar reflex with tapping, or cooperating with difficulty with loud verbal instructions; and 6 = sleeping, not cooperative), the amount of analgesia consumed, nausea and vomiting, and any side effects.

### 2.1. Statistical Analysis

Analysis of the data was conducted using SPSS 15.0 software. In the evaluation of the data, frequency distribution, mean, standard deviation, percentages, and cross-tabulation were used. Categorical comparisons were made using chi-square or Fisher's exact tests. In groups with homogenous distribution, the Kolmogorov-Smirnov *z*-test was used; to determine a difference between groups, the Kruskal-Wallis or one-way ANOVA was applied. To determine from which group the difference originated, the Tukey HSD and Dunnett tests were applied. A value of *p* < 0.05 was accepted as statistically significant.

Sample size was measured with G^*∗*^Power using 3.0.10 statistical package programme. For 0.85 power (1 − *β*), effect size = 0.4, *α* = 0.05, minimum total patient number was found to be *n* = 72 (for each group *n* = 24). A total of 75 patients were enrolled (*n*1 = 25, *n*2 = 25, and *n*3 = 25). After we completed the study we measured the power of the study with G^*∗*^Power using 3.0.10 statistical package programme. 0.87 power was found for (1 − *β*), effect size = 0.4, and *α* = 0.05 (*n*1 = 25, *n*2 = 25, and *n*3 = 25).

## 3. Results

No statistically significant difference was found between the groups in terms of demographic data (age, height, weight, gender, ASA distribution, anesthesia, and surgery duration) (Table 1, *p* > 0.05).

**Figure 1:**
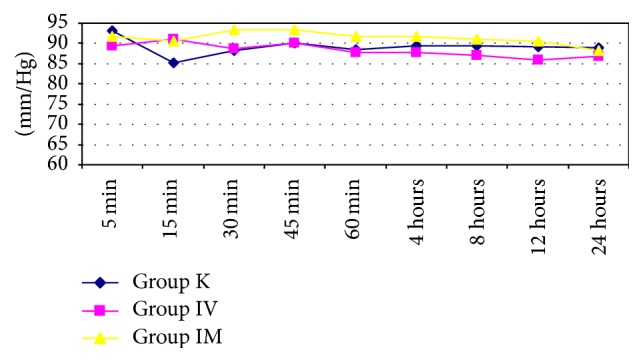
Measurements taken from 5 minutes to 24 hours postoperatively for mean arterial pressure between the groups.

**Figure 2 fig2:**
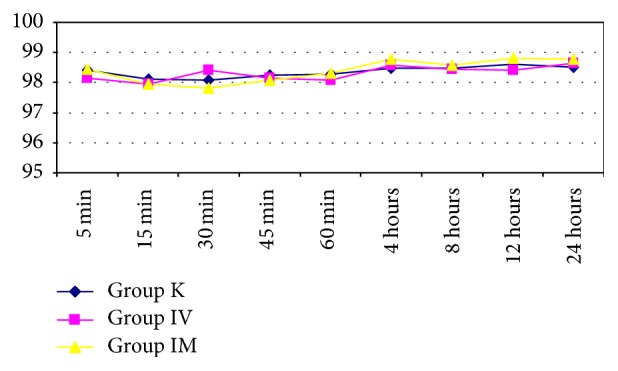
Measurements taken from 5 minutes to 24 hours postoperatively for SpO_2_ values between the groups.

**Figure 3:**
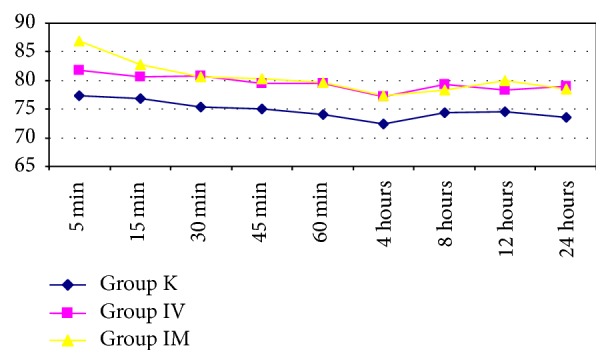
Measurements taken from 5 minutes to 24 hours postoperatively for heart rate values between the groups.

In the comparisons between the groups, no statistically significant difference was found between the groups with the measurements taken from 5 minutes to 24 hours postoperatively for mean arterial pressure, SpO_2_, and heart rate (Figures 1 [Fig fig2]–3, *p* > 0.05).

In the comparison of tramadol consumption between the groups measured at 5, 15, and 60 minutes, the mean value of the IM Group was statistically significantly lower than that of Group C (*p* < 0.05) and no difference was found between Group C and the IV Group or between the IM Group and the IV Group. In the comparison of tramadol consumption between the groups measured at 30 and 45 minutes, the mean value of the IM Group was significantly lower than that of Group C and the IV Group (*p* < 0.05); no difference was found between Group C and the IV Group. In the comparison of tramadol consumption between the groups measured at 4, 8, 12, and 24 hours, the mean value of the IM Group was significantly lower than that of Group C and the IV Group (*p* < 0.05); the IV Group was found to be significantly lower than Group C (Table 2, *p* < 0.05).

A 75 mg dose of diclofenac sodium was administered intramuscularly as additional analgesia to 3 patients in Group C, 2 patients in the IM Group, and 2 patients in the IV Group.

No statistically significant difference was found between the groups at the times measured from 5 minutes to 24 hours postoperatively in terms of the Numeric Rating Scale and the Ramsay Sedation Scale (Table 3, *p* > 0.05).

Nausea and vomiting side effects occurred at a rate of 36% in Group C, which was a significantly higher rate compared to the other groups (Table 4, *p* < 0.05).

## 4. Discussion

In this study the effects of magnesium sulfate administered systemically or to the muscles in the surgical area intraoperatively on postoperative tramadol consumption were investigated in patients undergoing lumbar disc surgery under general anesthesia. While the total postoperative tramadol consumption of the Control Group was found to be high compared to the IV Group and the IM Group, the tramadol consumption of the IM Group was lower than that of the IV Group.

Magnesium is used in several clinical practices in anesthesia and pain syndromes [16, 17]. Intravenously administered magnesium in spinal, epidural anesthesia and axillary brachial plexus blocks has been shown to reduce postoperative analgesia consumption [18–20]. There are few studies comparing the effects of magnesium applied systemically or to the surgical area on postoperative analgesia. In radical prostate surgery, magnesium applied as an infusion to the skin incision has been shown to reduce postoperative tramadol consumption more than intravenously applied magnesium [20]. This localized effect of magnesium is explained by the synergistic interaction between peripheral NMDA receptor blockage and ropivacaine.

It is thought that magnesium has no systemic effect; the fact that the serum magnesium level after magnesium administration is lower than the serum magnesium level before administration may be due to magnesium absorption. In a study of arthroscopic knee surgery, a significant reduction in total analgesic consumption was observed in the group to which intra-articular bupivacaine-magnesium solution was applied compared to groups that received only magnesium and bupivacaine [21]. The effect of magnesium here is explained by its effect being increased by delaying bupivacaine absorption pharmacodynamically in addition to the pharmacological effect of the receptor mechanisms. In another study, intra-articular magnesium alone was administered to one group while intra-articular saline was given to another group and the postoperative analgesic effects were observed. The analgesic requirement of the magnesium group was found to be lower than that of the saline group [22]. When all these studies and results are taken into consideration, the significant reduction in the need for tramadol following magnesium applied to the surgical area without adding local anesthetic compared to systemically administered magnesium demonstrates the strength of the peripheral effect of magnesium.

The reduction in pain with the administration of intravenous magnesium can be explained by central sensitization and NMDA receptor blockage in the spinal cord. The effect of magnesium applied to the surgical area is not yet fully understood. This effect may be partially explained by the fact that, in addition to the central localization of NMDA receptors, receptors have been found in the periphery (muscle, skin, and knee joint) that have a role in the transfer of pain stimuli [10, 11] or the antagonizing of the expression of inflammatory mediators (histamine, serotonin, and cytokines) in the peripheral tissue [7]. There remains a need for studies investigating the effect of magnesium applied to the surgical area on pain pathways.

In the current study, additional analgesia was required by 3 patients in Group C and 2 patients each in the IM Group and the IV Group. The NRS was higher in Group C than in the IM and IV Groups in the first 12 hours but the difference was not statistically significant. The NRS values of the IM and IV Groups were similar. This can be explained by the patients using the PCA effectively. Medications adjuvant to analgesia are expected to decrease the need for additional analgesia without affecting sedation or increasing the side effect profile. The findings of the current study show that magnesium met these expectations. The sedation scores were similar in all the groups. In addition to nausea and vomiting, no other side effects were observed. Nausea was observed in 9 patients in Group C, in 5 in the IV Group, and in 3 in the IM Group. Vomiting was observed in 9 patients in Group C, 4 patients in the IV Group, and 3 patients in the IM Group. This is thought to be related to the reduced consumption of tramadol. Similar to the findings of the current study, Kara et al. found lower rates of postoperative vomiting in the magnesium group but the difference was not statistically significant [14]. A limitation of the current study was that the serum magnesium level was not measured.

In conclusion, magnesium administered directly to the surgical area was found to be more effective than systemically administered magnesium in achieving postoperative analgesia without causing any side effects. However, there is a need for further studies to investigate the mechanism of the analgesic effect of peripherally applied magnesium.

## Figures and Tables

**Table 1:** Demographic characteristics (mean ± SD, *n*), anesthesia, and operation duration (mean ± SD).

	Group C (*n* = 25)	IV Group (*n* = 25)	IM Group (*n* = 25)
Age (years)	46.36 ± 12.43	43.76 ± 10.55	42.16 ± 8.86
Weight (kg)	72.80 ± 6.36	75.28 ± 11.07	77.08 ± 10.65
Height (cm)	167.68 ± 7.77	169.20 ± 9.31	169.48 ± 8.50
Gender (F/M)	12/13	11/14	13/12
ASA (I/II)	11/14	13/12	12/13
Anesthesia duration (mins)	122.00 ± 19.37	127.80 ± 22.13	125.20 ± 23.56
Operation duration (mins)	111.12 ± 19.23	118.08 ± 21.53	115.24 ± 22.60

No statistically significant difference was found (*p* > 0.05).

**Table 2:** Comparison of tramadol consumption (mean ± SD).

	Group C (*n* = 25)	IV Group (*n* = 25)	IM Group (*n* = 25)	*p*
5 mins	11.52 ± 7.33^*∗*^	10.24 ± 7.84	5.76 ± 7.84^*∗*^	**0.025**
15 mins	22.40 ± 10.33^*∗*^	18.56 ± 8.86	12.16 ± 9.56^*∗*^	**0.001**
30 mins	32.64 ± 9.78^*∗*^	28.16 ± 10.61^*∗*^	19.20 ± 12.22^*∗*^	**0.000**
45 mins	46.08 ± 10.65^*∗*^	41.60 ± 14.61^*∗*^	30.08 ± 15.54^*∗*^	**0.000**
60 mins	60.16 ± 11.57^*∗*^	53.12 ± 14.40	44.80 ± 29.57^*∗*^	**0.031**
4 hours	164.48 ± 43.94^*∗*^	126.08 ± 49.60^*∗*^	88.32 ± 40.56^*∗*^	**0.000**
8 hours	266.24 ± 65.46^*∗*^	195.20 ± 65.65^*∗*^	139.52 ± 53.37^*∗*^	**0.000**
12 hours	298.72 ± 57.65^*∗*^	247.68 ± 62.87^*∗*^	199.76 ± 59.80^*∗*^	**0.000**
24 hours	335.72 ± 59.09^*∗*^	283.68 ± 64.61^*∗*^	240.76 ± 61.19^*∗*^	**0.000**

^*∗*^Statistically significant (*p* < 0.05).

**Table 3:** Comparison of the numeric rating scores (mean rank mean ± SD).

	Group C (*n* = 25)	IV Group (*n* = 25)	IM Group (*n* = 25)	*p*
5 mins	45.32 (5.63 ± 1.48)	37.76 (4.88 ± 1.86)	32.83 (3.84 ± 1.64)	0.063
15 mins	44.94 (5.44 ± 1.33)	37.94 (4.68 ± 1.75)	33.12 (3.76 ± 1.51)	0.072
30 mins	44.50 (4.44 ± 1.16)	39.28 (4.20 ± 1.78)	34.22 (3.44 ± 1.39)	0.088
45 mins	43.50 (3.96 ± 1.14)	35.88 (3.44 ± 1.47)	32.62 (3.24 ± 1.23)	0.093
60 mins	45.06 (3.60 ± 1.08)	34.82 (2.96 ± 1.27)	32.72 (2.80 ± 1.19)	0.068
4 hours	40.12 (2.40 ± 1.15)	35.30 (2.12 ± 1.33)	38.58 (2.28 ± 1.31)	0.709
8 hours	33.80 (1.44 ± 1.04)	39.66 (1.76 ± 1.16)	40.54 (1.80 ± 1.22)	0.468
12 hours	36.26 (1.20 ± 1.08)	40.22 (1.48 ± 1.33)	37.52 (1.20 ± 0.87)	0.786
24 hours	31.66 (0.32 ± 0.69)	41.70 (0.96 ± 1.51)	40.64 (0.60 ± 0.71)	0.121

No statistically significant difference was found (*p* > 0.05).

**Table 4:** Comparison of complications (*n*/%).

	Group C	IV Group	IM Group	*p*
	(*n* = 25)	(*n* = 25)	(*n* = 25)	
Vomiting				
No	16 (64.0%)	21 (84.0%)	22 (88.0%)	**0.040**
Yes	^*∗*^9 (36.0%)	4 (16.0%)	3 (12.0%)	
Nausea				
No	16 (64.0%)	20 (80.0%)	22 (88.0%)	**0.044**
Yes	^*∗*^9 (36.0%)	5 (20.0%)	3 (12.0%)	

^*∗*^Statistically significant (*p* < 0.05).
